# Statistical fallacies in orthopedic research

**DOI:** 10.4103/0019-5413.30524

**Published:** 2007

**Authors:** Abhaya Indrayan

**Affiliations:** Department of Biostatistics and Medical Informatics, University College of Medical Sciences, Delhi - 110 095, India

**Keywords:** Biased sample, differential definition, misuse of means, inadequate analysis, misuse of *P-* values, medical significance

## Abstract

**Background:**

A large number of statistical fallacies occur in medical research literature. These are mostly inadvertent and occur due to lack of understanding of the statistical concepts and terminologies. Many researchers do not fully appreciate the consequence of such fallacies on the credibility of their report.

**Materials and Methods:**

This article provides a general review of the issues that could give rise to statistical fallacies with focus on orthopedic research. Some of this is based on real-life literature and some is based on the actual experiences of the author in dealing with medical research over the past three decades. The text is in teaching mode rather than research mode.

**Results:**

Statistical fallacies occur due to inadequate sample that is used for generalized conclusion; incomparable groups presented as comparable; mixing of two or more distinct groups that in fact require separate consideration; misuse of percentages, means and graphs; incomplete reporting that suppresses facts; ignoring reality and depending instead on oversimplification; forgetting baseline values that affect the outcome; misuse of computer packages and use of black-box approach; misuse of *P*-values that compromises conclusions; confusing correlation with cause-effect; and interpreting statistical significance as medical significance.

**Conclusion:**

Mere awareness of the situations where statistical fallacies can occur may be adequate for researchers to sit up and take note while trying to provide a credible report.

Fallacies are anomalies that considerably reduce the credibility of a report. Statistical fallacies are common in medical research literature. This article enumerates a large number of such fallacies, particularly in orthopedic research, in the hope of creating awareness about situations where these can occur. Such awareness by itself may be enough for a researcher to try to produce a more credible report.

Statistics show that more people die in hospital than at home. Also, there is a strong association between dying and being in bed. The nonsense of such associations is apparent. No one would advocate avoiding a hospital or bed to prolong life! These might seem like extreme examples, but the same sort of errors of logic sometimes pass unrecognized in medical literature.^1^

Abuse or misuse of statistics has precisely the same consequence. An *abuse* occurs when the data or the results are presented in a distorted form with the intention of misleading. Sometimes part of the information is deliberately suppressed to support a particular hypothesis. A *misuse* occurs when the data or the results of analysis are unintentionally misinterpreted due to lack of comprehension. The fault in either case cannot be ascribed to statistics. It lies with the user. The difficulty, however, is that the adverse effects of wrong biostatistical methods are slow to surface. And that makes these methods even more vulnerable to wrong uses.

## PROBLEMS WITH THE SAMPLE

The subjects of investigation should provide sufficient and valid data to take a decision one way or the other. Sometimes this does not happen for a variety of reasons.

### Biased sample

A sample is considered unbiased when it truly represents the target population. A frequent source of error in statistical conclusions is a biased sample. This can happen even when the selection is random or even when random allocation is made in experimental studies. A large sample tends to magnify these errors rather than to control them.

#### Survivors:

Consider the relationship of bone mineral density (BMD) with age in women after the age of 60 years. It is well known that this density declines due to hormonal changes but the gradient differs from population to population. How do we find the exact effect of age in a particular population? Those with lower density are likely to be in poor health and thus may have less longevity. Any estimation based on survivors is bound to be biased in this situation. It may not be easy to find the correct gradient in this case.

Similarly, a study based only on hospital cases will exclude those who die before admission. Serious cases, those residing in remote areas and those who are poor to afford hospitalization tend to be excluded. Similar bias occurs in a variety of other situations where the study is on prevalent cases rather than on incident cases. Both these fallacies may have occurred in investigating age influence on the ability of femoral BMD to predict hip fracture where the subjects were women aged 75 and over.^2^

#### Volunteers:

Early phases of clinical trials are in any case done on volunteers. Volunteers tend to be very different from the general class of subjects. Many of them are either hopeless terminal cases or are subjects with exceptional courage. Both affect the response. Notwithstanding this limitation, volunteer studies have a definite place in medicine as they do provide important clues on the toxicity of the regimen under test, the dose level that can be tolerated and the potential for further testing of the modality.

Medical ethics require that the subject's consent must be taken for the research. Real consent, after fully explaining the underlying uncertainties, is difficult to obtain. Many researchers in developing countries just get the consent form signed, which a sick person may do under the force of circumstances, without properly understanding the consequences. When the real consent is taken, the subjects again self-select themselves and the sample may become biased. Some of this is eliminated if and when a random allocation strategy is adopted but that is feasible only for trials. Even then generalizability suffers. Conclusions based on all such research can be misleading. Not many researchers appreciate this fallacy and tend to wonder why their results do not apply to the practical situation.

#### Clinic subjects:

Clinic and hospital subjects too form a biased sample, as they tend to have a more severe form of disease and include mostly those who can afford these services. Mild cases tend to ignore their condition or self-treat themselves. An interesting example is migraine, which was once believed to be more common in the intelligent professional class.^3^ An epidemiological study on a random sample could not substantiate such a relationship when the role of confounding factors was eliminated. Those in the more intelligent professional class perhaps seek medical assistance in the early phases of the disease and with greater frequency than their nonprofessional counterparts. Despite such limitations, clinic-based studies do give important information on the presenting symptoms, their correspondence with laboratory and radiological findings, response to various therapeutic procedures, prognostic features, etc. But the results are seldom applicable to the type of cases that do not show up in clinics. It is sometimes believed that consecutive cases coming to a clinic would be free from bias. Such cases could be truly representative of clinic subjects, but the general bias in all clinic-based subjects still remains.

#### Inadequate size of the sample:

Statisticians are notorious for advising large samples. The number of subjects in a study should be adequate to generate sufficient confidence in the results. Considering this aspect, a larger sample is not harmful but a small sample can be a waste of resources. A small sample may fail to detect the differences really present in the target population. There is a growing concern among the medical community regarding the failure of many randomized controlled trials (RCTs) in detecting a clinically relevant difference because they do not have sufficient power. The driving force for power is the size of the trial and the inter-individual variability. In only 3% studies published in the year 1997 in American and British volumes of Clinical Orthopedics and Related Research, the power was adequate to detect a small difference.^4^

In addition, a small sample has high likelihood of being not fully representative and thus of producing biased results. Statistical methods have an inbuilt provision to take care of the larger sampling fluctuations in small samples but they are not equipped to take care of the lack-of-representativeness bias that is more likely to creep into small samples.

In a rare situation, an exceedingly large sample could also be a problem. Schnitzer *et al*^5^ studied prescribing pattern for rofecoxib and colecoxib in 47,935 patents of osteoarthritis and 10,639 patients of rheumatoid arthritis. The objective was to find the most frequently prescribed dose. They realized the futility of the statistical test of significance since clinically unimportant small differences would turn out to be statistically highly significant for such a large sample. Despite such a big sample, their conclusion had limitations due to lack of clinical information, inability to ascertain actual use and potential for selection bias.

#### Nonresponse:

Statistical nonresponse occurs when some cases are lost to follow-up. A large nonresponse can render an otherwise good sample ineffective in drawing a valid conclusion not only because of the bias it tends to introduce but also because it considerably reduces the size of the sample available for inference. See, for example, the study on quality of life after hemiarthroplasty for hip fracture by Poulain *et al*.^6^ where 18.7% cases were lost to follow-up and another 18.2% died. Missing values, if not far too many, can be handled by imputation methods, although this involves a complex statistical procedure. But this cannot reproduce the sample. Thus, efforts must be made at the stage of planning and at the stage of data collection to ensure that the nonresponse is minimum.

#### Publication bias:

Meta analysis that tends to provide more valid and reliable conclusions by pooling results from different studies is surely an accepted statistical technique. However, two points deserve attention in this regard. First, literature is generally unduly loaded with “positive” results. Negative or indifferent results are either not sent for publication or are not published by the journals with the same frequency. Any conclusion based on commonality in publications thus can magnify this bias.

It must be realized that many consistent results increase confidence but do not necessarily provide confirmatory evidence. A single contradictory finding with credence can put a question mark on the whole conclusion.

### Incomparable groups

If sufficient care is not exercised with regard to various features of the groups under comparison, fallacies can easily occur. For comparability, the control should undergo the same clinical maneuvers, such as transfer from one unit to another and duration and frequency of examination, which are used for the treatment group. This is what makes blinding so important. If a group of women receiving arthroscopic surgery of the knee is being observed for deep venous thrombosis, the same set of observations should be done on the women with other knee surgeries for a valid conclusion regarding excess incidence in the arthroscopic group. In practice, the group of women with other knee surgeries may not be observed for thrombosis with similar attention.

#### Differential in group composition:

Matching or randomization is advised as a strategy to minimize bias in results based on comparison of groups. Comparability should not be merely for age and gender but should be for all those prognostic factors also that might possibly affect the outcome. These include severity of disease, coexisting diseases, care of the subjects, etc. This equivalence is difficult to achieve in practice. Also, all prognostic factors may not be fully known. Randomization is a good strategy to average out the factors and to obtain two equivalent groups, and it works well in the long run. It may fail in a particular study. It is necessary that similarity of groups is checked as a posthoc procedure even when the groups are formed by genuine randomization. This similarity has to be cautiously interpreted because a large difference could be statistically not significant if the sample size is small.

In a trial for performance of a new design trochanteric Gamma nail in comparison to compression hip screws in trochanteric fractures,^7^ patients were randomized but no specific matching was done for the number of stable and unstable fractures. The study did find that postoperative walking ability was better in patients with unstable fractures when treated with the nail. Perhaps such a trial should be carried out separately for patients with stable and unstable fractures. In this study, they were mixed and separated afterwards for the purpose of analysis that might have affected the validity of the conclusion.

#### Differential definitions:

In a study on the trend in contribution of osteoporosis to total morbidity over the past 50-60 years, an important factor is the change in the definition and detection of osteoporosis from 1950 to today. The disease is much better understood now than 50 years ago. The diagnostic procedures have greatly improved after the emergence of DEXA and the awareness is high. Thus, the detection rate has increased. This will surely affect the trend.

Defining osteoporosis as BMD at least 2.5SD below the normal for 25-year-old females^8^ implies under Gaussian conditions that six in 1000 ‘healthy’ 25-year-old females would be osteoporotic! In fact these may be much less since they are healthy. Such an anomaly exists with all statistical definitions that identify the level below which (or above which) the risk dramatically increases. Most medical measurements follow a continuum and a valid cutoff is difficult to identify. Thus fallacies occur and they are tolerated.

#### Differential compliance:

In a clinical trial setup, subjects in the treatment group may drop out more because of discomfort or poor taste of the drug, even when the placebo looks like the drug and the trial is randomized. On the other hand, the subjects in the treatment group may stay if they see improvement in their condition whereas the placebo group can become noncompliant. The compliance rate in this case is related to the efficacy of the regimen and the comparison can be jeopardized.

A related situation occurs when different groups do not have the same opportunity of being screened just as we mentioned earlier on thrombosis in arthroscopic surgery. Diabetes and gallstones may appear to be associated because diabetics are regularly checked for gallstones but the nondiabetics are rarely checked.

#### Improper denominator:

If the distribution of ankylosing spondylosis in different blood groups is O, 45%; A, 35%; B, 15%; and AB, 5%, it would be naive to conclude that this disease occurs more commonly in subjects with blood group O. For a correct conclusion, this distribution should be compared with the proper denominator, which in this case is the blood group distribution in the concerned population.

Another kind of problem with the denominator occurs when organs or episodes are counted instead of individuals. Modern medicine indeed seems to have fragmented human being into a conglomerate of different parts-organs, tissues, etc. An orthopedic surgeon may count the average number of vertebrae in a group of cases of spinal tuberculosis^9^ or number of knees^10^ or number of replaced hips.^11^ Although this might be adequate in some instances, it may be inadequate to assess the magnitude of the problem. Organs belonging to the same person are not statistically independent as they are across individuals.

A similar problem arises when a subject is repeatedly counted in case of recurrent episodes. This can happen, for example, for low back pain.^12^ Recurrent attacks tend to have same origin. Extra caution may be required in stating and interpreting results based on such repeated count of individuals.

### Mixing of distinct groups

Mixing of distinct groups can give fallacious results in some situations. Two are illustrated next.

#### Effect on regression:

**Figure 1:**
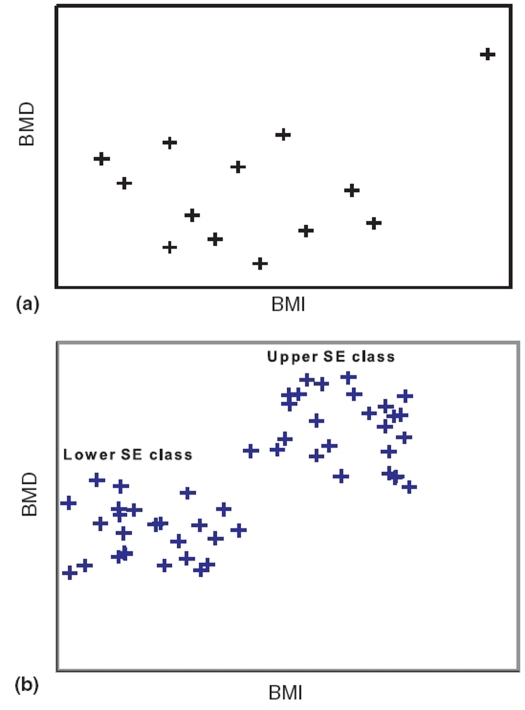
Relationship with outlier and when two groups, each with no relationship, are merged

An annoying feature of regression (and correlation) is that it can be influenced by a single outlier. The position is shown in Figure 1a. If the outlier is excluded, there is no relationship but if it is included it can produce a fairly high degree of relationship.

Mixing of two distinct groups can give a false sense of regression. Consider a survey on boys of Grade IX belonging to three randomly selected schools from each of the two strata-schools in slums catering to a low socioeconomic (SE) class and schools in posh localities catering to an upper class. The objective was to find health correlates. Among the measurements made were body mass index (BMI) and BMD in left wrist. When these are plotted, a scatter of the type shown in Figure 1b is obtained. This contains two distinct clusters of points, the lower left belonging to the low SE class and the upper right belonging to the upper SE class.

The points within each cluster are randomly scattered and indicate no relationship whatsoever between BMD and BMI in either class. That is, there is no evidence that a high BMI in either class is accompanied by a high value of BMD. When the data for the two SE classes are mixed, a distinct relationship emerges. But that is false because neither group has that kind of relationship unless one wants to draw a composite conclusion for the two groups combined.

## ERRORS IN PRESENTATION OF FINDINGS

Out of ignorance or deliberately, the presentation of data in medical reports sometimes lacks propriety. This can happen in a variety of ways.

### Misuse of percentages

Percentages can mislead if calculations are (a) based on small *n* or (b) based on an inappropriate total. If two patients out of five respond to a surgery, is it correct to say that the response rate is 40%? In another group of five, if three respond, the rate jumps to 60%. A difference of 20% looks like a substantial gain, but in fact the difference is just one patient. This can always occur due to sampling fluctuation. Thus, percentage based on small *n* can mislead. Suppose 200 subjects are asked about their preference for more convenient, less expensive, but less efficacious medical treatment versus less convenient, more expensive, but more efficacious surgical treatment. Only 60 (30%) reported preference for medical intervention and 84 (42%) preferred surgery. The remaining 56 (28%) were noncommittal. If this finding is reported as 42% preferring surgery, keeping silent about the others, it has the risk of being interpreted as saying that the remaining 58% preferred medication. This obviously is wrong. The nonrespondents or the neutrals should always be stated so that no bias occurs in the interpretation.

If 12% of critical patients in the control group die within one week and 8% in the treatment group, the risk reduction is 4% or 33%? Results are sometimes stated to magnify the effect without the corresponding explanation regarding the base of calculation.

### Misuse of means

A popular saying by detractors of statistics is, “Head in an oven, feet in a freezer and the person is comfortable!” There is no doubt that an inference on mean alone can sometimes by very misleading particularly when it is based on just two values as in this case. Mean should not be calculated unless there are at least four readings. In addition, it must always be accompanied by the standard deviation (SD) so that an indication is available about the dispersion of the values on which the mean is based. In the quoted phrase, the SD would be exceedingly high showing that the mean is useless. Sometimes, the standard error (SE) is stated in place of SD but that too might mislead unless its implications in the context are fully explained. Also, *n* must always be stated when reporting a mean. These two, *n* and SD, should be considered together when drawing any conclusion based on mean. Statistical procedures such as confidence intervals and test of significance have a built-in provision to take care of both of them. A mean based on large *n* naturally commands more confidence than the one based on small *n*. Similarly, a smaller SD makes the mean more reliable.

General practice is to state mean and SD with a ± sign in between such as mean BMD in lumbar spine in healthy premenopausal women is 0.98±0.075 g/cm^2^.^13^ Opinion is now generating against the use of the ± sign because it tends to give a false impression that the variation is from 0.905 to 1.055 g/cm^2^ in this case. The variation is much more-even more than ±2SD limits, 0.830 to 1.130 g/cm^2^. Thus it is better to state clearly that mean is 0.980 and SD is 0.075 g/cm^2^ without using a ± sign.

Mean may not be an appropriate indicator for a particular data set. If in a group of eight persons, seven do not take alcohol and one consumes 200 mL per day, how correct is it to say that the average consumption in this group is 25 mL per person per day? If extreme values or outliers are present, mean is not a proper measure. Either use the median or recalculate mean after excluding the outliers. If exclusion is done, this must be clearly stated.

### Misuse of graphs

Some fallacies can occur due to inadequate choice of scale in a graph. A steep slope can be represented as mild and vice versa. Similarly, a wide scatter may be shown as compact. Also, means in different groups or means over time can be shown without corresponding SDs. They can be shown to indicate a trend that does not really exist or is not statistically significant.

One of the main sources of fallacies in graphs is their insensitivity to the size of *n*. A mean or a percentage based on *n* = 2 is represented the same way as the one based on *n* = 100. The perception, and possibly cognition, received from a graph is not affected even when *n* is explicitly stated. One such example is the box-and-whiskers plot drawn for the time elapsed between cancer and acquired immunodeficiency syndrome (AIDS) diagnoses among homosexual men with cancer diagnosed before or concurrently with AIDS in San Francisco during 1978 to 1990,^14^ where *n* is 4 in one group yet maximum, minimum, first quartile, third quartile and median are shown.

### Problems in Reporting

Among many problems that can occur with the reporting, two requiring specific attention are incomplete reporting and overreporting.

#### Incomplete reporting:

All reports should state not only the truth but the whole truth. There is a growing concern in the medical fraternity that part of the information in reports is sometimes intentionally suppressed and sometimes unknowingly missed. If so, the reader gets a biased picture. This is easily illustrated by the bias of many medical journals for reporting of “positive” findings and ignoring the “negative” reports. Both should be reported in a balanced manner. Similarly, properly designed studies that do not reject a particular null hypothesis deserve a respectable place in the literature. That, at present, is sadly lacking although awareness for a balanced approach is increasing. Recently a Journal of Negative Results in Biomedicine has started to partly address this problem.

A very serious problem is reporting only that part of a study that supports a particular hypothesis. The other parts are suppressed. An example is a series of studies on the carcinogenic effect of asbestos. According to an analysis of different studies, deliberate attempts were made by the industry to suppress information on the carcinogenicity of asbestos that affected millions of workers.^15^

#### Over reporting:

The statement of results in a report should generally be confined to the aspects for which the study was originally designed. If there are any unanticipated findings, these should be reported with caution. These can be labeled as “interesting” or “worthy of further investigation” but not presented as conclusions. A new study, specifically designed to investigate these findings, can then be conducted.

#### Self-reporting versus objective measurement:

Self-perception of health may be very different from an assessment based on measurements. A person with an amputated leg may consider himself absolutely healthy. This can particularly happen with social and psychological domains of quality of life. Besides such discrepancies, it has been observed, for example, that people tend to report lower than actual weight but higher height. This could make a substantial difference when BMI is calculated. The percentage of subjects with BMI^3^ 25 may thus become much lower than obtained by actual measurements. Thus, only those characteristics should be self-reported that are so required for the fulfillment of the objectives of the study. All others should be objectively measured.

## INADEQUATE ANALYSIS

Among the most common sources of fallacies in data-based conclusions is the use of inappropriate method of analysis.

### Ignoring reality

Most statistical methods take a simplified view of the complex biological process. Because computers are available for intricate calculations, the statistical need to simplify is really not as great now as it used to be in the precomputer era. But simplification is still required for easy comprehension. This should not be done to an extent that could distort the essential features of a biological process.

#### Looking for linearity:

There is no doubt that hardly any relationship in medicine is linear. Yet, linear relationship is the most commonly studied form of relationship in health and medicine. This simplification seems to work fairly well in many situations but can destroy an otherwise very clear relationship in others. The rise and fall of lung function with increase in age is aptly represented by a parabolic curve but the relationship vanishes if only linearity is considered. Another example is the relationship between glomerular filtration rate and creatinine level. Kidney function can influence bone health in at least older people^16^ and needs to be properly studied. This linear relationship is medically unsatisfactory in this example even if *R*^2^ is high.^17^ There is a definite need to curb the tendency to linearize a clear nonlinear relationship.

Statistical linearity also includes a parabola with square term of the regressor because the coefficient is still linear. McQuellon *et al*^18^ found that overall quality of life trajectory in bone narrow transplant recipient is parabolic. This shows that terms such as square and log should be included where indicated by scatter plot.

#### Assumptions overlooked:

A Gaussian form of distribution of various quantitative measurements is so ingrained in the minds of some workers that they take it for granted. For large *n*, the central limit theorem can be invoked for inference on means but nonparametric methods should be used when *n* is small and the distribution is far from Gaussian. At the same time, note also that most parametric methods, such as *t* and *F*, are quite robust to mild deviation from the Gaussian pattern. Their use in such cases does limited harm so long as the distribution has a single mode.

Transformations such as logarithm and square-root sometimes help to “Gaussianize” a positively skewed distribution. But these also make interpretation difficult and unrealistic. If the logarithm of blood lead concentration is weakly correlated with log of bone lead concentration,^19^ what sort of conclusion can be drawn for the lead concentration itself? Despite this limitation, such transformations are in vogue and seem to lead to correct conclusions in many cases, particularly if *n* is not too small. The assumptions of independence of observations and of equality of variance across groups are more important than that of a Gaussian pattern. Independence is threatened when the measurements are serial or longitudinal. Uniformity of variance is lost when, for example, SD varies with mean. And this is not so uncommon. For example, as the cell-densities of cultured osteoblast life MG63 cells increase, their SDs also increase.^20^ Persons with higher BMI also tend to exhibit greater variability in BMI than those with lower BMI. Thus, care is needed in using methods that require uniformity of variance such as ANOVA *F*-test.

In the case of Chi-square for proportions, the basic assumption is that the expected frequency in most cells is five or more. This restriction is sometimes overlooked. Fisher's exact test for a 2×2 table has become an integral part of most statistical packages of repute but the multinomial test required for larger tables with expected frequencies less than five has not found a similar place.

#### Anomalous person-years:

It is customary to calculate person-years of follow-up and use this for various inference purposes. Person-years of exposure is a valid epidemiological tool only when each year of exposure has the same risk. Calculation of the mortality rate per thousand person-years after fracture in patients on dialysis^21^ presumes that the risk of mortality in the first year after fracture is the same as in, say, the tenth year after fracture. This obviously is not true. If nothing else, ageing will make an impact in this case.

### Mean or proportion?

The blame lies mostly with statisticians who fancy quantity than quality. Although quantitation in many cases does help in achieving exactitude in thinking and in conclusions, it can sometimes suppress important findings.

Consider a rise in BMD in lumbar spine after a high single dose of vitamin D (60,000 IU) orally in 10 children of age 5 to 13 months diagnosed as nutritional rickets.^22^ The authors have not given the exact values but suppose they were as follows:

BMD before the drug (g/cm^2^)

0.25 0.23 0.19 0.27 0.20 0.32 0.24 0.30 0.24 0.26

BMD after one month of single dose of vitamin D (g/cm^2^)

0.28 0.29 0.27 0.27 0.28 0.31 0.28 0.29 0.29 0.24

Increase (g/cm^2^)

0.03 0.06 0.08 0.00 0.08 -0.01 0.04 -0.01 0.05 -0.02

Mean increase from 0.25 g/cm^2^ to 0.28 g/cm^2^ in this example is statistically significant by paired *t*-test (*P* = 0.034). However, the increase occurred in six cases and not in the other four cases. A marginal fall in BMD one month after the dose can occur in some cases due to biological variation or instrumentation. If the dose is not effective, the probability of increase in BMD is the same as the probability of decrease. Under this null hypothesis (H_0_: *P* = ½), the exact probability of decrease in three or less subjects out of 10 by binomial is 0.17. This is more than 0.05. Thus the null cannot be rejected.

The proportion gives a negative result about the effect of the dose in this example whereas the mean difference gives positive result. Depending upon the preference of the investigator, the finding can be presented either way.

### Forgetting the baseline values

The conclusion in the preceding example is based on the absolute increase in BMD in different subjects. Critical examination of the data reveals that increase occurred mostly in the subjects with low baseline values. The pattern generally is lower the baseline BMD, higher the rise. The above analysis based first on paired *t*-test and second on binomial ignores this important aspect. The conclusion in this example is that dose generally helps nutrition rickets children with BMD≤0.025 g/cm^2^. This can be statistically established by running a regression.

### Misuse of statistical packages

Computers have revolutionized the use of statistical methods for empirical inferences. Methods requiring complex calculations are now done in seconds. This is a definite boon when appropriately used but is a bane in the hands of nonexperts. Understanding of the statistical techniques has not kept pace with the spread of their use, particularly in medical and health professionals.

#### Overanalysis:

Data are sometimes overanalyzed, particularly in the form of post hoc analysis. A study may be designed to investigate the relationship between two specific measurements but correlations between pairs of a large number of other variables which happen to be available, are calculated and examined. If each correlation is tested for statistical significance at *α* = 0.05, the total error rate increases enormously. Also, *α* = 0.05 implies that one in 20 correlations can be concluded to be significant when it actually is not. If measurements on 16 variables are available, the total number of pair-wise correlations is 16×15/2 = 120. At the error rate of 5%, six of these 120 can turn out to be falsely significant. Hofacker^23^ illustrated this problem with the help of randomly generated data. He suggests that the data on half the subjects be kept aside for use in a validation exercise. This is feasible when the data for a large number of subjects are available but not otherwise. There is a tendency to find the age-sex or severity group that benefited more from the treatment than the others. Numerous such analyses are sometimes done in the hope of finding some statistical significance somewhere.

#### Data dredging:

Because of the availability of computer packages, it is now easy to reanalyze data after deleting some inconvenient observations. Valid reasons for this exercise, such as outliers, are sometimes present, but this can be misused by excluding some data that do not fit the hypothesis of the investigator. It is extremely difficult to get any evidence of this happening in a finished report. Integrity of the workers is not and cannot be suspected unless evidence to the contrary is available. Thus, data dredging can go unnoticed.

#### Quantitative analysis of codes:

Most computer programs, for the time being, do not have the capability to distinguish numeric codes from quantitative data. If disease severity is coded as 0, 1, 2, 3, 4 for none, mild, moderate, serious and critical conditions respectively, statistical calculations may treat them as the usual quantitative measurements. This runs the risk of considering three mild cases equal to one serious case and so on. That is, codes can be mistreated as scores. This can happen even with nominal categories such as signs and symptoms when coded as 1, 2, 3, etc. Extra caution is needed in analyzing such data so that codes do not become quantities.

## MISINTERPRETATION

Misinterpretation of statistical results mostly occurs due to failure to comprehend them in their totality and inability to juxtapose them with the realities of the situation. This can happen either because of the limited knowledge of many medical professionals about statistical concepts^24^ or because of inadequate understanding of medical issues by the statisticians associated with medical projects.

### Misuse of *P*-values

Statistical *P*-values seem to be gaining acceptance as a gold standard for data-based conclusions. However, biological plausibility should not be abandoned in favor of *P*-values. Inferences based on *P*-values can also produce a biased or incorrect result.

### ‘Magic’ threshold 0.05:

A threshold 0.05 of Type I error is customary in health and medicine. Except for convention, there is no specific sanctity of this threshold. A result with *P* = 0.051 is statistically almost as significant as one with *P* = 0.049, yet the conclusion reached would be very different if *P* = 0.05 is used as the threshold. Borderline values always need additional precaution.

The practice now generally followed is to state exact *P*-values so that the reader can draw his own conclusion. A value of *P* around 0.10 can possibly be considered weak evidence against the null hypothesis and a small *P*, say less than 0.01, as strong evidence. This way it acquires continuum.

### One-tail or two-tail *P*-values:

If a regimen can do no harm, such as iron supplementation for augmentation of low Hb level, one-tail test is appropriate. Use of two-tail test in this case makes it unnecessarily restrictive and makes rejection of H_0_ more difficult. Most statistical packages provide two-tail *P*-values as a default and many workers would not worry too much about this aspect. Scientifically, a conservative test does not do much harm, although some real differences may fail to be detected when a two-tail test is used instead of a one-tail test. Our advice is to use a one-tail test wherever clear indication is available but not otherwise.

### Dramatic *P*-values:

Attempts are sometimes made to dramatize the *P*-values. James *et al*^25^ stated *P* < 0.000,000,000,01 for difference in seroconversion rate against Epstein-Barr virus between lupus patients and controls. Such accuracy is redundant. It really does not matter whether *P* < 0.001 or *P* < 0.000,001 as far as its practical implication is concerned.

### ‘Conclusion’ with respect to several parameters:

Consider individual persons instead of groups. The reference range for most quantitative medical parameters is obtained as mean ± 2SD of healthy subjects. These statistical limits carry a risk of excluding 5% healthy subjects who have levels in the two extremes. When such limits are applied on several parameters, it becomes very unlikely that all parameters in any person are within such statistical range - even if he is fully healthy. This anomaly is sometimes forgotten while devising inclusion and exclusion criteria for subjects in the study.

This fallacy is similar to multivariate conclusion based on several univariate analyses and the one inherent in multiple comparisons. When the study is based on more than two groups, the Type I error swells when each group is compared with each of the others. To control this, methods such as Tukey and Dunnett are used. Few trials in surgery and medicine consider such adjustment for multiple comparison.^26^ Multivariate conclusion refers to joint conclusion on the basis of several outcomes. The same sort of increase in Type I error occurs in this case. A similar problem occurs when, for example, Student's *t*-test is used to compare osteoblast-like cells at several points of time when exposed and unexposed to a static magnetic field.^20^ When each comparison is made at 5% level of significance, the total Type I error for all comparison together could be unbearably large.

#### Correlation versus cause-effect relationship

An association or a correlation in health can arise due to a large number of intervening factors. It is rarely a cause-effect type of relationship unless established by a carefully designed study that rules out the possibility of a significant role of any confounding factor.

A strong correlation between heights of siblings in a family exists not because one is the cause of the other but because both are affected by parental height. Similarly, correlation between visual acuity (VA) and metacarpal index in subjects of age 50 years and above is not cause-effect type but arises because both are products of the same degeneration process. We do not expect VA to improve if metacarpal index is improved by some therapy.

An unusual confounding factor may provide useful information in some cases. Comparison of surgical and nonsurgical treatment of nondisplaced acute scaphoid fractures^27^ thankfully considered poverty as a confounding factor, where the outcome is time taken to return to active work. Poverty can force people to return early but is many times forgotten as a confounding factor in such studies.

Distinction may be made between a necessary cause and a sufficient cause. Sexual intercourse is necessary for initiating pregnancy but it is not sufficient. In fact, the correlation between the number of intercourses and number of pregnancies, even without barriers, is negligible.

### Sundry issues

The list of statistical fallacies seems to never end. Some of those not discussed above are as follows.

#### Medical significance versus statistical significance:

An improvement of one point in quality of life after a surgery can be statistically significant if *n* is large but may not have any medical significance in terms of condition of the patient or in terms of management of the condition. Statistical methods check statistical significance only and medical significance needs to be examined separately.

The other potentially hazardous error is in interpreting statistical nonsignificance (failure to reject H_0_) as insignificance. A very important difference between two groups may be present but it will be statistically not significant if *n* is small or if the variability between the subjects who happened to be in the samples is high. The null hypothesis cannot be rejected unless there is sufficient evidence against it. Thus, conceding a null should not be implied to mean that the difference is not present. Nonsignificance is not necessarily the equivalent of ‘no difference”.

#### Limitation of relative risk:

No risk can exceed one. Five-year risk of death in leukemia may be 0.97 and in arthritis only 0.02. Then the relative risk (RR) of death in leukemia is nearly 48. Opposed to this, if the comparison is with bone cancer cases where the five-year risk of death is 0.70, the RR is only 0.97/0.70 = 1.4. This is nearly as high as it can get in this situation. Note the physical limitation imposed by high risk in the baseline group. This aspect is many times forgotten while interpreting the value of relative risk.

## LAST WORD!

No decision is more important than the one concerning the life and health of people. The medical fraternity has a tall order. They are expected to prolong life and reduce suffering that occurs as a consequence of complex and often poorly understood interaction of a large number of factors. Some of these factors are explicit but many remain obscure and some behave in a very unpredictable manner. Uncertainties in health and medicine are indeed profound. Knowingly for some but unknowingly for many, statistical methods play a vital role in all empirical inferences. Statistical methods can be a dangerous tool when used carelessly. Computer-based statistical packages have not yet been given expertise to decide the correct method although they sometimes generate a warning message when the data are not adequate. The user of the package decides the method. If you are not sufficiently confident, do not hesitate to consult an expert biostatistician.

Medical journals too have a responsibility to ensure that the results of dubious quality are not published. Statistical refereeing is a norm for some journals but some are lax on this issue.

Our last advice is not to rely solely on statistical evidence. Statistical tools are surely good as an aid but rarely as a master. Depend on your intuition more than science. If scientific results fail intuitional judgment, look for gaps. They would most likely lie with 'science' than with intuition.
